# Characterization of Micro-RNA Changes during the Progression of Type 2 Diabetes in Zucker Diabetic Fatty Rats

**DOI:** 10.3390/ijms17050665

**Published:** 2016-05-03

**Authors:** Denis Delic, Claudia Eisele, Ramona Schmid, Gerd Luippold, Eric Mayoux, Rolf Grempler

**Affiliations:** 1 Department of Translational Medicine and Clinical Pharmacology, Boehringer Ingelheim Pharma GmbH & Co. KG, 88397 Biberach an der Riss, Germany; denis.delic@boehringer-ingelheim.com (D.D.); claudia.eisele@boehringer-ingelheim.com (C.E.); ramona.schmid@boehringer-ingelheim.com (R.S.); 2 Department of CardioMetabolic Diseases Research, Boehringer Ingelheim Pharma GmbH & Co. KG, 88397 Biberach an der Riss, Germany; gerd.luippold@boehringer-ingelheim.com (G.L.); eric.mayoux@boehringer-ingelheim.com (E.M.)

**Keywords:** ZDF rats, hyperinsulinemia, diabetes, micro-RNA, biomarker, disease progression, miR-122, miR-133a, miR-375

## Abstract

The aim of the present pilot study was the identification of micro-RNA changes over time during the development and progression of type 2 diabetes (T2D) in Zucker diabetic fatty rats (ZDF rats). T2D is a complex metabolic disorder that is characterized, *inter alia*, by progressive failure of pancreatic β cells to produce insulin, but also by functional or morphological modifications of others organ, such as liver, adipose tissue and the cardiovascular system. Micro-RNAs are a novel class of biomarkers that have the potential to represent biomarkers of disease progression. In this study, the onset and progression of diabetes was followed in ZDF rats from six weeks until 17 weeks of age. After an initial phase of hyperinsulinemia, the animals developed T2D and lost the capacity to produce sufficient insulin. Circulating miRNAs were measured from plasma samples at four time points: pre-diabetes (six weeks of age), hyperinsulinemia (eight weeks), β cell failure (11 weeks) and late-stage diabetes (17 weeks) using TaqMan miRNA arrays. Bioinformatic analysis revealed distinct changes of circulating miRNAs over time. Several miRNAs were found to be increased over the course of the disease progression, such as miR-122, miR-133, miR-210 and miR-375. The most significantly decreased miRNAs were miR-140, miR-151-3p, miR-185, miR-203, miR-434-3p and miR-450a. Some of the miRNAs have also been identified in type 2 diabetic patients recently and, therefore, may have the potential to be useful biomarkers for the disease progression of T2D and/or the treatment response for anti-diabetic medications.

## 1. Introduction

Type 2 diabetes (T2D) affects 285 million people worldwide (2010), and the numbers are projected to increase substantially in the years to come up to 366 million in 2030 [1,2]. Several effective drugs are available for the treatment of T2D with the generic metformin, sulfonylureas and thiazolidinediones and novel drugs, like Dipeptidyl peptidase-4 inhibitors (DPP-4i), Sodium-dependent glucose transporter inhibitors (SGLT2i), injectable Glucagon-like peptide-1 analogues (GLP-1a) or insulin. Despite the plethora of anti-diabetic medications, none of the available treatments is able to stop the progressive decline in pancreatic β cell mass and function and thereby stop the progression of T2D [3]. Because of this, many patients require combination treatment to prevent deterioration of glucose control over the years and later complications of T2D, like diabetic retinopathy or diabetic nephropathy [4]. In order to show the beneficial effects of new treatments for T2D on disease progression, clinical trials need to be performed over a long period of time up to five years of treatment. Early biomarkers that are sensitive and specific to predict the progression of T2D in patients are needed. Therefore, better knowledge in understanding the pathophysiological mechanisms will be crucial for designing new strategies to prevent and/or treat the progression of T2D.

Micro-RNAs (miRNAs) are small, 18–22 nucleotides in length, non-coding RNA molecules that modulate the differentiation, growth, apoptosis and proliferation of cells by interfering with protein synthesis by either inducing mRNA degradation or repressing translation [5,6]. Circulating miRNAs are remarkably stable due to resistance to degradation from endogenous RNase activity [7,8,9]. Several miRNAs are involved in the regulation of β cell differentiation, function and mass [10]. In particular, the direct involvement of miR-375 in insulin secretion via targeting myotrophin (MTPN) expression [11,12] and glucose homeostasis via targeting phosphoinositide-dependent protein kinase 1 (PDK-1) is well described [13,14]. Increased pancreatic miR-375 expression was reported in T2D patients [15] and also in animal models, such as *ob*/*ob* mice [16]. There are several other miRNAs, such as miR-7, miR-124a, miR-9, miR-34a and miR-195, that play a role in the regulation of insulin secretion and β cell development [17]. Defective β cells can release miRNAs into the bloodstream following pathophysiological conditions. Recently, Kong *et al.* [18] demonstrated significantly higher levels of miR-34a, miR-9, miR-29a, miR-30d, miR-124a, miR-146 and miR-375 in sera of newly-diagnosed T2D compared to people with normal glucose tolerance or pre-diabetes. Furthermore, plasma miRNA profiling of T2D patients showed loss of endothelial miR-126, probably resulting in impaired peripheral angiogenic signaling [19].

Alterations of miRNAs within the islets have also been reported in many animal models, such as obese models for T2D using *ob*/*ob* mice [16], *db*/*db* mice [20] and high fed diet-induced rat models [21] or the non-obese pre-diabetic non-obese diabetic mice model (NOD mice) [22] and the spontaneous T2D model using Goto-Kakazaki rats [23]. Mostly, these changes were analyzed at individual time points and not during disease progression. Furthermore, chemical induced models using streptozotocin (STZ) are widely used as pancreas injury models [24], and miR-375 has been found increased upon induction of diabetes [11].

Despite the fact that type 2 diabetes is a complex disease, which is strongly influenced by the lifestyle of Western societies with high caloric diets and reduced physical activity, as well as underlying genetic factors, it seems that certain miRNAs are clearly associated with type 2 diabetes, and further research is required to understand the causes and consequences.

The Zucker diabetic fatty (ZDF) rat model has become a gold standard to monitor disease progression of T2D, as many characteristics of human conditions are reflected [25]. The ZDF rats are characterized by a defect in the leptin pathway, which results in metabolic syndrome, obesity and T2D in early stages of development. The ZDF rat is an ideal model to observe the natural progression of T2D from initial hyperinsulinemia to failure of β cells and loss of insulin production and then progressive diabetes in aging animals. However, changes in miRNA expression profiles in plasma of ZDF rats during disease progression have never been explored.

In the present study, we aimed to identify changes in the plasma miRNA profile of ZDF rats during the natural progression of T2D. Most of the altered circulating miRNAs reflect changes in diabetes-related tissues, such as pancreas, liver, heart, skeletal muscle and adipose tissues. These miRNAs could potentially serve as prognostic markers of disease progression and potentially as treatment effect markers in T2D.

## 2. Results

### 2.1. Type 2 Diabetes Progression in ZDF Rats

**Figure 1:**
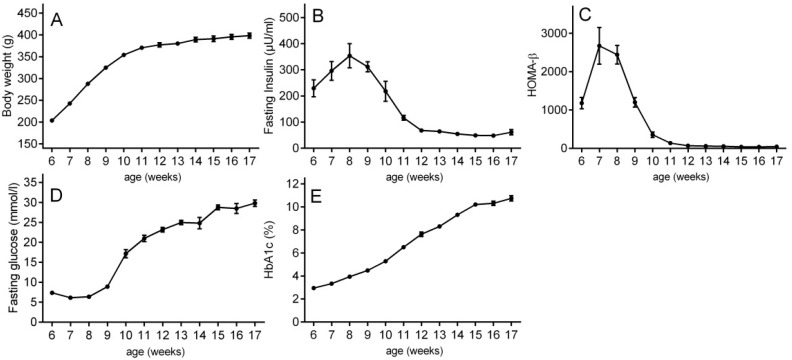
Development and progression of type 2 diabetes in ZDF rats from six weeks of age to 17 weeks of age. (**A**) Body weight; (**B**) fasting plasma insulin; (**C**) HOMA-β; (**D**) fasting plasma glucose; and (**E**) HbA1c were measured weekly following a 2-h food deprivation. Results are expressed as the mean ± SEM.

The present study characterizes the micro-RNA changes during natural progression of T2D in chow-fed, aging ZDF rats from six weeks of age to 17 weeks of age (*n* = 10). The body weight of ZDF rats increased from 6–12 weeks of age and reached a plateau around 12 weeks (Figure 1A). The ZDF rats showed initial fasting hyperinsulinemia at eight weeks of age to compensate for the increasing peripheral insulin resistance (Figure 1B). At around nine weeks of age, the capacity of β cells to secrete more insulin started to decline (Figure 1B). This decline in fasting insulin secretion continued until 11–12 weeks of age, when the β cells finally failed to secrete sufficient amounts of insulin and the animals became fully hyperglycemic and diabetic. The described course of β cell decompensation was further confirmed by HOMA-β, an index of β cell function (Figure 1C). Fasting hyperglycemia increased in parallel to the loss of insulin secretion from week 9–12 up to approximately 25 mM fasting glucose (Figure 1D). HbA1C levels rose steadily from week 6 over the entire course of the study (Figure 1E). Due to these results, the present study is ideally suited to characterize the longitudinal miRNA profile at different disease stages from (A) pre-diabetes (PD) (six-week-old ZDF rats) to (B) hyperinsulinemia (HI) (at eight weeks of age) to (C) β cell failure (BCF) (at 11 weeks of age) to (D) late-stage diabetes (LSD) (at 17 weeks of age).

### 2.2. Discovery of miRNA Profiles during Diabetes Disease Progression

For the discovery of free circulating micro-RNA in the plasma from the ZDF rats, plasma samples from the pre-diabetes group and from animals at the disease stages hyperinsulinemia, β cell failure and late-stage diabetes were used. All results were normalized to the respective pre-diabetes time point for the same group of animals. The analysis was performed as described in the Materials and Methods, and the results were subjected to bioinformatic assessment.

### 2.3. Bioinformatic Assessment of miRNA Changes in Plasma Samples of ZDF Rats

**Figure 2:**
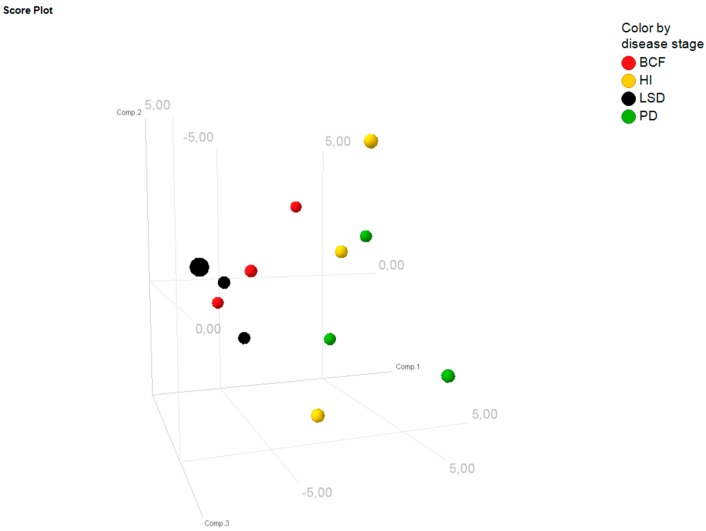
Principal component analysis. Displayed are the first three major components from the principal component analysis for each replicate per time point: PD = pre-diabetes; HI = hyperinsulinemia; BCF = β cell failure; LSD = late-stage diabetes.

Principal component analysis (PCA) revealed that the overall expression profiles differed among the four different time points of disease progression (Figure 2). In contrast, the expression profiles were relatively similar among the three biological replicates, which indicate a significant change of miRNAs over the disease course and the consistency of the results at each time point. Overall, the PCA indicates a gradual change of miRNA levels during disease progression (Figure 2).

**Figure 3:**
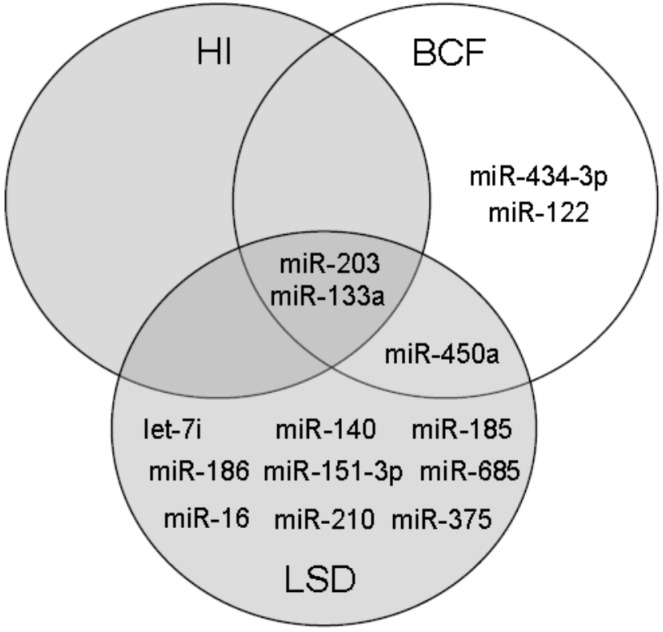
Venn diagram. Numbers of altered miRNAs at hyperinsulinemia (HI), β cell failure (BCF) and late-stage diabetes (LSD) are summarized. Overlaps represent those miRNAs that are significantly changed at more than one time point.

**Figure 4:**
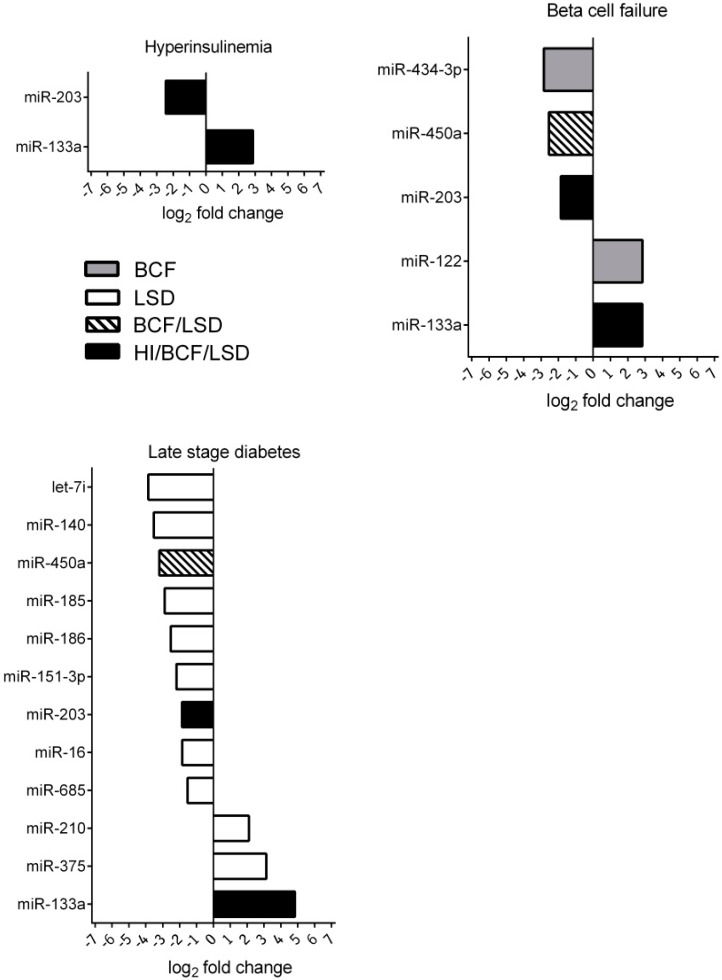
Significantly-altered circulating miRNAs (fold-change >2; *p* < 0.05) at individual time points during disease progression at hyperinsulinemia (HI), β cell failure (BCF) and late-stage diabetes (LSD) analyzed by qRT-PCR. Log_2_-fold changes of increased or decreased miRNA species at HI, BCF and LSD are indicated. Each time point was compared to the baseline (week 6 of age).

**Figure 5:**
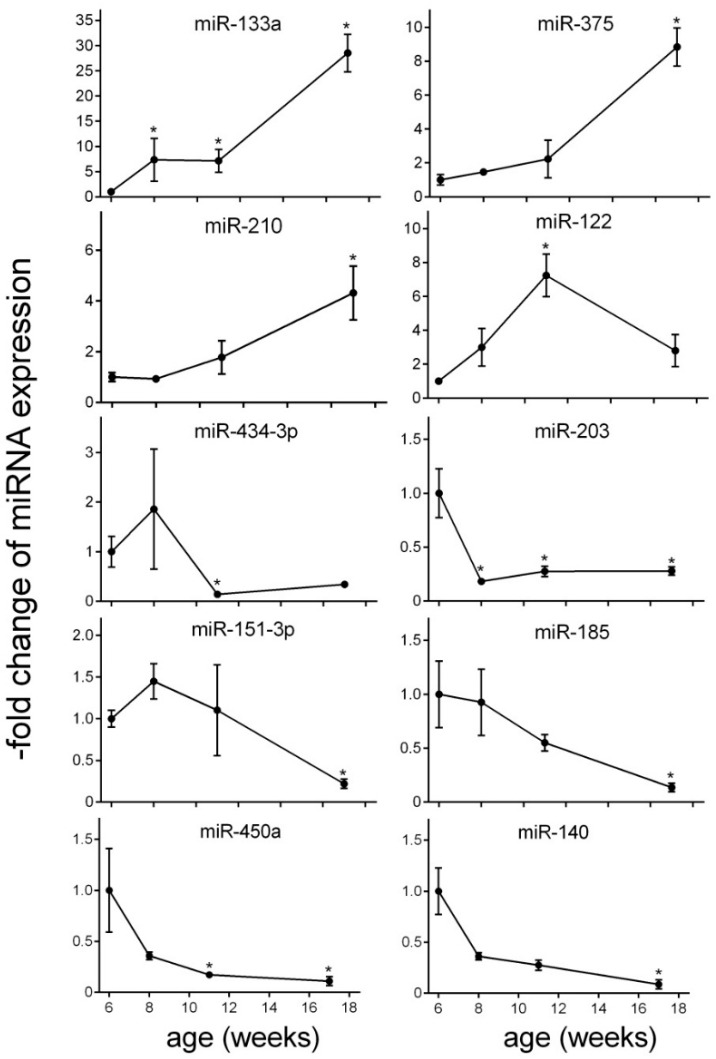
Significantly-altered miRNAs during disease progression. Relative expression is given as the fold increase to baseline (PD). Significant differences to baseline are indicated by * (*p* < 0.05). Values represent the mean ± SEM.

The total number of detected circulating miRNAs in plasma samples was 111. The numbers of altered circulating miRNAs at each time point are summarized in the Venn diagram (Figure 3). For the initial analysis, group-wise comparisons were performed for each time point to assess the significant changes compared to baseline (fold-change >2 and *p* < 0.05) (Figure 4). At initial hyperinsulinemia, the circulating level of miRNA-133 was significantly increased compared to the pre-diabetes stage, whereas the level of miR-203 was decreased (Figure 4 or Figure 5). When β cell failure occurred, the circulating level of miR-133a remained high, and additionally, the level of miR-122 was significantly increased, whereas miR-203, miR-450a and miR-434-3p were decreased at this time point (Figure 4). miRNAs, such as miR-434-3p and miR-122, were specifically changed at the time point of β cell functional failure (Figure 4). At late-stage diabetes, the circulating level of 12 miRNAs was specifically altered; the circulating level of miR-375, miR-210 and miR-133a was increased, and the circulating levels of let-7i, miR-140, miR-450a, miR-185, miR-186, miR-151-3p, miR-203, miR-16 and miR-685 were strongly diminished *versus* their levels at the pre-diabetes stage (Figure 4). Two miRNAs were significantly altered across all disease stages. The expression of miR-133a was persistently increased, and the level of miR-203 was persistently decreased (Figure 4).

### 2.4. miRNA Changes during Disease Progression in ZDF Rats

In order to characterize the changes of circulating miRNA level over time during disease progression, an ANOVA was performed (*F*-statistic *p*-value < 0.05) (Table 1, Figure 5 and Figure S1). Overall, the blood level of 3 miRNA species is significantly elevated, namely miR-133a, miR-375 and miR-210. The highest augmentation of miRNA blood level was observed for miR-133a, which was approximately 28-fold higher at late-stage diabetes than at the pre-diabetes stage (Figure 5). Overall, a gradual elevation of circulating levels of miR-133a was observed over the disease course. The expression of miR-122 represented an exception reaching a peak already at β cell functional failure (approximately seven-fold elevation). Diminution in circulating levels was detected for miR-140, miR-151-3p, miR-185, miR-203, miR-434-3p and miR-450a (Figure 5). The blood level of miR-140 was more than 12-fold suppressed at late-stage diabetes (Figure 5). The profile of the circulating level of miR-185, miR-450a, miR-203 and miR-140 was characterized by a continuous decline over time compared to pre-diabetes. The miR-151-3p level were slightly increased at initial hyperinsulinemia and decreased at late-stage diabetes (Figure 5). The drop of miR-203 was already detected at hyperinsulinemia (Figure 5), and the level of this miRNA remained low at all time points compared to pre-diabetes. The level of miR-434-3p was reduced at BCF and LSD. The functions of the miRNAs based on published data and their experimentally-observed and/or predicted main target mRNAs are summarized in Table 1.

## 3. Discussion

The present study characterizes the changes in the free circulating level of micro-RNAs in blood during the natural progression of T2D in chow-fed, aging ZDF rats from pre-diabetes over initial hyperinsulinemia to β cell failure and late-stage diabetes. The rats show initial hyperinsulinemia at eight weeks of age to compensate for the insulin resistance. Then, the insulin secretion declined until 11–12 weeks of age, when the β cells finally fail to secrete sufficient amounts of insulin and the animals become fully hyperglycemic and diabetic, also represented by HOMA-β. During the progression of T2D, the numbers of significantly altered miRNA species are constantly increased from two miRNAs at HI (week 8 of age) to five miRNAs at BCF (week 11 of age) to 12 miRNAs at LSD (week 17 of age), indicating physiological responses to diabetic complications.

The circulating level of 10 miRNAs displayed significant alterations during the progression of the disease (Table 1). In general, the false discovery rate of the described 10 miRNAs in this study is between 1% and 3.5% (Table 1), and therefore, the identified miRNAs are very unlikely false positives.

Most of our identified plasma miRNAs are known to be modified in tissues known to play a role in diabetes, such as pancreas, liver, skeletal muscle and adipose tissues or in the cardiovascular system.

Based on previous work, the expression of the two increased miRNA species miR-375 and miR-210 and the three decreased miRNAs, miR-203, miR-185 and miR-450a, is involved in the regulation of insulin secretion and pancreatic β cell function [11,26,27,28]. Our study revealed a continuous elevation of circulating miR-375 levels. miR-375 is abundantly expressed in β cells and serves as a negative regulator of insulin secretion via targeting myotrophin expression [11]. Another important target of miR-375 is PDK1 in β cells, which results in a decrease of glucose regulation [13]. Recently, it was demonstrated that circulating miR-375 levels in mice only originated in a small proportion from β cells, but mainly from various neuroendocrine cells from lung, gastrointestinal tract, thyroid and adrenals [29]. This indicates that this miRNA is not a specific marker for β cell function in mice. Nevertheless, a markedly increased circulating miR-375 level in response to streptozotocin, indicating acute β cell injury, was described several times [24,29]. Therefore, the strong augmentation of miR-375 blood level (>8-fold) at late-stage diabetes in our study may still reflect ongoing β cell death. One limitation of our study is that we do not have serial tissue biopsies to compare the miRNA changes with histological changes, e.g., of the pancreas. Altered miR-375 level does not exclusively reflect T2D pathophysiology. Recently, it has been shown that miR-375 expression is decreased in pancreatic cancer, and overexpression of miR-375 impacts cell proliferation and apoptosis in pancreatic cancer cells [30,31]. Therefore, we can only hypothesize that miR-375 could reflect progressive β cell death. The repeated identification of miR-375 in diabetes models and the apparent high expression in β cells demands a further qualification of this potential type 2 diabetes progression biomarker in animals and, potentially, human studies. It needs to be determined whether miR-375 changes in plasma reflect expression changes of the miRNA in the pancreas at a constant rate of β cell death or if miR-375 reflects indeed the apoptosis of β cells.

miR-203 levels were strongly decreased at all time points during the progression of the disease. Therefore, miR-203 might be considered as an early indicator of type 2 diabetes onset in ZDF rats. In a recent study, decreased miR-203 and miR-210 expressions were detected in pancreatic islets of young pre-diabetic and diabetic *db*/*db* mice and in mice fed with a high fed diet [26] and were strongly associated with β cell dysfunction [20,22]. Therefore, a similar regulation of miR-203 was observed in *db*/*db* mice and high fat diet-fed mice (HFD mice) compared to our study. The study of Nesca *et al.*, 2013, observed a decrease in miR-210 in pancreatic islets [26]. Our study revealed a continuous elevation of miR-210 blood level in ZDF rats. The observed increase in blood might be a result of modifications in organ function or morphology as increased circulating miR-210 levels were detected, e.g., under hypoxic conditions in cardiac tissue, and miR-210 expression increases during myocardial infarction in other studies [32].

Furthermore, our study showed significant alteration in the circulating level of miR-185 and miR-450a during the progression of the disease. While the role of miR-450a on the regulation of endocrine function or in diabetes is not clear to date, miR-185 plays an important role in the regulation of insulin secretion and β cell growth via targeting suppressor of cytokine signaling 3 (SOCS3) [27]. A significantly decreased miR-185 blood level was detected in patients with diabetes and in pancreatic islets of diabetic mouse models [27]. These findings point towards a functional role of miR-185 in diabetes progression. However, miR-185 can also block cardiac hypertrophy signaling and was discussed as a target for heart failure treatment [33]. Furthermore, miR-185 has been identified in several cancer types and may therefore not be a specific marker. Further elucidation of the role of miR-185 in humans is required.

The miR-133a displayed the strongest elevation during disease progression in our study. miR-133a is abundantly expressed in skeletal muscle and myocardial cells [34,35]. Overexpression of miR-133 in cardiomyocytes reduced GLUT4 expression and insulin-stimulated glucose uptake by targeting KLF15. Therefore, miR-133 may play an important role in the pathogenesis of insulin resistance in ZDF rats [36]. In addition, circulating plasma miR-133a levels are increased in patients with acute myocardial infarction and potentially serve as a marker for cardiomyocyte death [37]. It has been shown that aging ZDF rats have altered left ventricular chamber morphology and function at 16 weeks of age [38]. Therefore, miR-133 may be a potential biomarker to follow the consequences of disease progression at the level of skeletal muscle or cardiac muscles.

The miR-151-3p level was decreased at late-stage diabetes in ZDF rats. In a recent study, downregulation of miR-151-3p was demonstrated during later stages of transverse aortic constriction-induced cardiac hypertrophy in mice, reflecting its participation in heart dysfunction [39]. In our study, both the diminution of miR-151-3p and the strong increase in miR-133a level at late-stage diabetes could therefore be the consequence of heart muscle dysfunction.

At β cell failure, circulating miR-434-3p levels were strongly decreased. Due to high variability at HI, no conclusion can be drawn about the early time point. In aged mouse skeletal muscle, miR-434-3p levels were found to be decreased [40] and in ApoE-deficient mice during the disease progression of atherosclerosis [41]. Our findings suggest a role of miR-434-3p during the progression of type 2 diabetes in ZDF rats. However, the mechanisms behind this reduction are elusive and require further investigation.

Our miRNA profiling study revealed that miR-122 expression is increased in plasma of ZDF rats during disease progression of T2D. miR-122 is considered to be a highly sensitive and specific biomarker in blood, reflecting hepatocyte injury, as increased levels of miR-122 were described in various liver diseases, such as non-alcoholic steatohepatitis (NASH) [42]. In NASH patients and in animal models, circulating miR-122 levels are elevated in serum, whereas its expression is decreased in liver tissue [43,44]. The strong increase of miR-122 is consistent with the fatty liver that ZDF rats develop when reaching their body weight plateau [45].

The strongest diminished miRNA in blood was miR-140, reaching an approximately 12-fold reduction at late-stage diabetes. Decreased miR-140 levels are associated with impaired regulation of osteogenesis and adipogenesis [46]. Adipocyte differentiation is regulated by miR-140 via targeting the TGF-β signaling pathway. Diminution of circulating miR-140 might be the result of an upregulated TGF-β signaling pathway during disease progression. The decrease of miR-140 at initial hyperglycemia might correlate with the dysfunctional adipocytes in ZDF rats and the insulin resistance of ZDF rat adipocytes [47].

In recent years, many different profiling studies in humans and animal models have used various profiling platforms to detect miRNAs as potential biomarkers for the disease progression of type 2 diabetes. There are large numbers of altered miRNAs obtained from cultured cells, blood or tissues, which described potential early candidate markers of T2D [48,49,50]. The eight circulating miRNAs, miR-29a, miR-34a, miR-375, miR-103, miR-107, miR-132, miR-142-3p and miR-144, and the two tissue-specific miRNAs, miR-199a-3p and miR-223, were identified to be significantly altered in T2D across a meta-analysis of controlled profiling studies [51]. Apart from miR-375, none of these miRNAs were strongly and significantly changed in our study. However, for instance, miR-29a was found to be decreased during disease progression in our study, as expected, but did not reach the level of statistical significance. Therefore, both miR-29a and miR-375 could be assessed in future studies as potential translatable biomarkers. Some of the diabetes-associated miRNAs were below the detection limit in our study, such as miR-9, miR-96 and miR-148, indicating that back-translation from humans to ZDF rats may be difficult for these markers.

The assessment of circulating miRNAs has several analytical and technical challenges. Plasma samples can be contaminated with cell remnants form erythrocytes, leukocytes and platelets. Erythrocytes have high levels of miR-16 and miR-451, which correlate to the degree of hemolysis in plasma samples [52]. In particular, hemolysis affects miRNA quantification [52,53]. Due to the design of our study and the comparisons to the pre-diabetes time point, an influence of hemolysis on our results is not likely, but cannot be entirely ruled out. The inconsistent findings on possible associations of miRNA changes across the literature can also be partly caused by diverse normalization strategies, as normalization is a crucial step in any quantitative real-time PCR experiment [54,55]. In our study, global mean normalization was used, which is one of the most robust normalization approaches.

One limitation of the current study is that it does not include a ZDF lean control group to control for potential age-related miRNA changes in non-diabetic rats. We acknowledge the possibility that age-related changes could impact miRNA levels in plasma. However, it has to be taken into account that the life span of a normal rat is 1.5–3 years (78–156 weeks). In ZDF rats, the onset of diabetes occurs at a very young age, around 8–12 weeks. In our study, the rats were used until an age of 17 weeks, which corresponds to a young age in normal rats.

In principle, several reports have implicated miRNAs in cellular senescence and aging. Mostly, these changes were observed on tissue level [56]. The following miRNAs were found to be altered in aging rats mostly from 48 weeks–132 weeks of age in skeletal muscle, e.g., miR-22 [57], or in murine liver tissues, e.g., miR-669c and miR-709, miR-93 and miR-214 [58,59]. miR-34a is a potential plasma marker of brain aging identified in old mice, starting to increase approximately two-fold at an age of 48 weeks [60]. In addition, monitoring age-related miRNA in the circulation in humans revealed only modest fold changes, even when comparing to widely-separated groups of age. After multiple adjustments, plasma miR-126-3p was significantly higher, but less than two-fold elevated in the oldest healthy subjects (>75 years) compared to the youngest (<45 years) [61].

None of the above mentioned miRNAs were found to be significantly changed in the plasma of diabetic ZDF rats in the age range of 8–17 weeks in the current study. Taken together, it is not expected that the observed changes in plasma miRNAs in diabetic ZDF rats can be explained by senescence and aging.

The treatment effects of anti-diabetic drugs on miRNA expression are well described. Metformin leads to decreased circulating miR-140-5p (miR-140) and miR-122 levels in T2D patients [62]. In endothelial cells, the anti-angiogenic effect of miR-34a via targeting SIRT1 is modulated by metformin [63]. In diabetic nephropathy, the miRNA-29 family protects the kidney from fibrotic damage, and the DPP-4 inhibitor linagliptin has been shown to inhibit TGF-β-induced endothelial to mesenchymal transition (EndMT) by restoring the miRNA-29s’ levels [64]. Our study also revealed a suppressed miR-29a level during disease progression of T2D in ZDF rats, but this was not significant and, therefore, not included in our candidate list. Nevertheless, the miR-29 family might serve as a candidate for monitoring of treatment effects.

This pilot study provides a first insight into the altered circulating miRNA profile during the disease progression of ZDF rats, and the changes in the miRNA profile likely reflect pathophysiological complications in several tissues, such as pancreas, liver, skeletal muscle, cardiac muscle and adipose tissue. Further studies are needed to underline their potential as early treatment effect biomarkers, as previously described for miRNA-29s. These novel, non-invasive markers show promise as tools for the mechanistic investigation of early disease progression of T2D and potentially to monitor treatment effects. As miRNAs regulate a number of downstream targets, the identified changes may be able to additionally uncover disease mechanisms in the ZDF rat. Future studies should elucidate the functional implications of the observed plasma miRNA changes for each target tissue.

## 4. Materials and Methods

### 4.1. Animal Study

The longitudinal T2D progression study was performed at Bêtagenex in Laval, Québec, Canada. Male ZDF Lepr^fa^/Crl (6 weeks old; Charles River Laboratories; L´Arbrescle Cedex, France) were housed in groups of 2 under pathogen-free conditions, controlled temperature on a regular 12-h light-dark cycle and received standard chow (Harland 2018) and water *ad libitum*. Animal experiments were conducted at the Centre Hospitalier de l´Universitć de Montréal and in accordance with the Policies and Guidelines of the Canadian Council for the Protection of Animals. The study code was BTX-BO002. All animals had an acclimation period of 5 days prior to the study start. Animals in the study were from 6 weeks of age until 17 weeks of age (*n* = 10). Body weight, 2-h-fasted glycemia, insulinemia and HbA1c were measured at baseline and then weekly from plasma or blood samples. For this, blood was taken from the tail vein of non-anaesthetized rats. All blood draws were carried out at the same time of the day following a 2-h food deprivation. Fasting glucose and fasting insulin were used to calculate the homeostasis model assessment of β cell function (HOMA-β). HOMA-β was calculated as (20 × fasting glucose (μU/mL)/fasting glucose (mmol/L) − 3.5).

### 4.2. miRNA Assessment: Isolation and Analysis

For the micro-RNA discovery analysis, pooled plasma from the ZDF rat samples was investigated. In detail, three plasma samples from the pre-diabetes group (PD) (6-weeks-old ZDF rats) and from animals aged 8, 11 and 17 weeks were used. Pooling was done according to insulin levels at baseline in a manner that reflects the overall mean in each pool. miRNA extraction of 150 μL pooled plasma (*n* = 3 pooled samples per group) was performed using the miRNeasy Serum/Plasma Kit (Qiagen, Hilden, Germany).

miRNA samples were normalized to 3.5 ng/μL and reverse-transcribed using the Megaplex Primer Rodent Pool A and the Taqman^®^ Micro RNA Reverse Transcription Kit (Applied Biosystems by Thermo Fisher Scientific, Waltham, MA, USA) according to the manufacturer’s protocol. With content matched to Megaplex stem-looped Rodent RT primers, Megaplex PreAmp Rodent Pool A Primers are used for unbiased preamplification, as recommended by the vendor.

The expression of the miRNAs was screened using Taqman^®^ Fast Advanced MasterMix (Applied Biosystems) and the rodent Taqman^®^ miRNA Array, Card A V.2.0 (Applied Biosystems). The gene expression analysis was run on a SDS7900HT real-time PCR system (Applied Biosystems by Thermo Fisher Scientific); raw *C*_t_ (cycle threshold) values were calculated using the SDS software v2.4 (Applied Biosystems by Thermo Fisher Scientific) with automatic baseline and threshold settings. Data were normalized to global mean *C*_t_. miRNAs with *C*_t_ values >32/undetermined were excluded. At least two of three replicates had to fulfill this criterion to be considered in the relative expression computation (2^−∆∆*C*t^, comparative *C*_t_ method) [65].

### 4.3. Data Analysis

Data was analyzed using R Version 3.0.1 (R Core Team, 2013, Vienna, Austria). The Bioconductor package HTqSeq v1.16.0 [66] was used to QC the data. Data were normalized using global mean normalization [67], only taking into account miRNAs with *C*_t_ < 35 for global mean calculation. For further analysis, only those miRNAs with raw *C*_t_ < 32 in at least two samples of at least one group were considered. For differential expression analysis, limma v3.18.13 [68] was applied. Fold changes comparing week 8 *versus* baseline (week 6), week 11 *versus* baseline, as well as week 17 *versus* baseline are reported. *p*-values are the false discovery rate (FDR) adjusted as proposed by Benjamini and Hochberg [69]. miRNAs displaying an adjusted moderated *F*-statistic *p*-value ≤0.05 are considered significantly altered in the ANOVA (Figure 5). For group-wise comparisons, miRNAs are considered significantly changed if the respective fold change ≥2 and adjusted *p*-value ≤0.05 (Figure 4).

## Figures and Tables

**Table 1:** Pathophysiological roles of deregulated miRNAs.

miRNA	FC Week 2	*p-*Value	FC Week 5	*p-*Value	FC Week 11	*p-*Value	FDR	Function	Target mRNA	PMID
Increased miRNA level										
miR-133a	7.36	0.041	7.14	0.018	28.54	0.001	0.009	abundantly expressed in heart; regulates expression of GLUT4 by targeting KLF15 and is involved in metabolic control in cardiac myocytes	KLF15 IGF1R	19720047
miR-375	1.46	0.737	2.23	0.374	8.84	0.007	0.028	abundantly expressed in pancreas; involved in pancreas development and β cell function	MTPN PDK	15538371
miR-210	0.92	0.88	1.77	0.42	4.32	0.019	0.032	involved in β cell dysfunction	HIF1α	23842730
miR-122	2.99	0.37	7.24	0.015	2.79	0.104	0.029	liver-specific miRNA involved in cholesterol biosynthesis pathway; upregulated miRNA level in non-alcoholic steatohepatitis patients (NASH patients)	HMGCR AMPKα1	16258535
Decreased miRNA level										
miR-434-3p	1.86	0.639	0.14	0.018	0.34	0.118	0.013	decreased expression in aged mouse skeletal muscle; downregulated during progression of atherosclerosis	–	25063768 23422117
miR-203	0.18	0.009	0.17	0.018	0.28	0.015	0.013	downregulation of miR-203 results in increased β cell apoptosis	TNFα	23842730
miR-151-3p	1.45	0.737	1.1	0.853	0.22	0.034	0.029	regulates slow muscle gene expression	ATP2A2	25200835
miR-185	0.93	0.885	0.55	0.431	0.14	0.007	0.02	targets suppressor of cytokine signalling 3 (SOCS3) to inhibit β cell dysfunction in diabetes	SOCS3	25658748
miR-450a	0.36	0.359	0.17	0.016	0.11	0.006	0.013	involved in the insulin secretion pathway	CCKAR	–
miR-140	0.36	0.612	0.27	0.139	0.08	0.007	0.028	surgery-induced (but not diet-induced) weight loss led to a marked decrease of miR-140	SMAD3	23396142

FC = fold change; FDR = false discovery rate; PMID = PubMed Identifier.

## Supplementary Materials

Supplementary materials can be found at http://www.mdpi.com/1422-0067/17/5/665/s1.

## Abbreviations

ACE	Angiotensin-converting enzyme
ARB	Angiotensin receptor blocker
BCF	B cell failure
CAD	Coronary artery disease
EndMT	Endothelial to mesenchymal transition
FDR	False discovery rate
HI	Hyperinsulinemia
LSD	Late-stage diabetes
miRNA	Micro-RNA
MTPN	Myotrophin
NASH	Non-alcoholic steatohepatitis
PCA	Principal component analysis
PD	Pre-diabetes
PDK-1	Phosphoinositide-dependent protein kinase 1
SOCS3	Suppressor of cytokine signaling 3
T2D	Type 2 diabetes
ZDF	Zucker diabetic fatty rats
*C*t	Cycle threshhold
DPP-4i	Dipeptidyl peptidase-4 inhibitors
HFD-mice	High fat diet-fed mice
SGLT2i	Sodium-dependent glucose cotransporter-2 inhibitors
GLP-1a	Glucagon-like peptide-1 analogues
NOD mice	Non-obese diabetic mice
PMID	PubMed Identifier
