# Supplementary material for: Characterization of Micro-RNA Changes during the Progression of Type 2 Diabetes in Zucker Diabetic Fatty Rats
Source: Int J Mol Sci. 2016 May 3;17(5):665. doi: 10.3390/ijms17050665 (PMC4881491; doi:10.3390/ijms17050665)
Supplement: Supplementary file 1 [file ijms-17-00665-s001.pdf]

Int. J. Mol. Sci. **2016**, *17*, 665; doi:10.3390/ijms17050665

Supplementary Materials: Characterization of Micro-RNA Changes during Progression of Type 2 Diabetes in Zucker Diabetic Fatty Rats

Denis Delic, Claudia Eisele, Ramona Schmid, Gerd Luippold, Eric Mayoux and Rolf Grempler

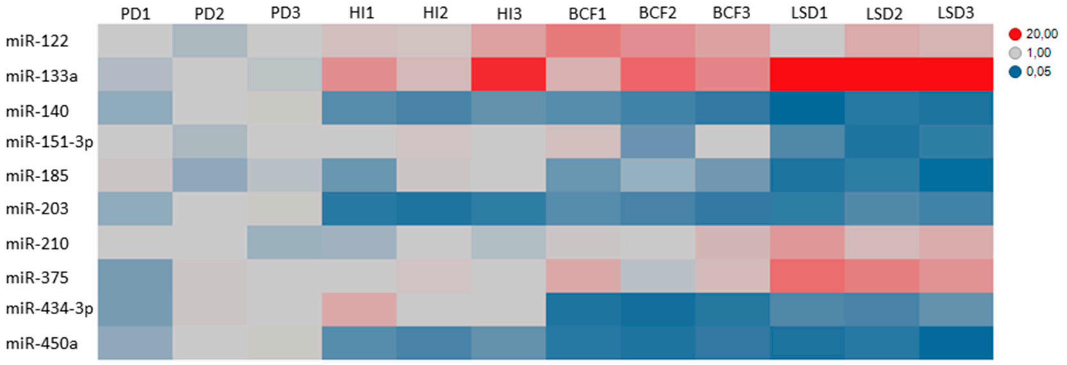

Figure S1. Heatmap of significantly altered miRNAs over the course of disease progression: pre-diabetes (PD), hyperinsulinemia (HI), β cell failure (BCF), and late-stage diabetes (**LSD**). Colors indicate increase (**red**; fold-change) or decrease (**blue**; fold-change) of miRNA levels compared to the pre-diabetes time point (Fold-change range from 20-fold increase (20) to 20-fold decrease (0.05)).
